# Benefits to Be Derived from Regular Medical Inspection of Our Schools

**Published:** 1913-08

**Authors:** G. M. Case

**Affiliations:** Elmira, N. Y.


### Benefits to be Derived From Regular Medical Inspection of Our Schools

BY G. M. CASE, M.D.
Elmira, N. Y.

[Read at the March, 1913, meeting of the Elmira Academy of Medicine]

THE authoritative school board, through legislative action, compels all children, between certain ages, to regularly attend school, and makes the term to last *practically* three-quarters of the year. Unless this is complied with the "delinquent" officer must know the reason. On the face of it this seems a just and commendatory measure, for, what is of more importance to the rising generation than a school education. But if the State had gone a step farther and made medical inspection of our schools compulsory, how much better it would have been for all concerned; for we medical men (and for that matter) most teachers know, that quite a large per cent. of the children in our schools need to have their bodies *treated* far more than to have their minds *trained*.

In fact, it is impossible to make much headway in training such children's minds until their physical condition is bettered. Every teacher encounters such a class of school children. They cannot make their grades, always behind in their studies, cannot apply themselves, and are, many times, ostracised and designated stupid. Nine times out of ten these children need medical attention. Perhaps their eyes are wrong, or they may be suffering from impaired hearing, or their mental faculties are dulled from obstructions in the upper air passages. Also, many of this class are improperly nourished, anaemic; may be suffering from digestive disorders or even incipient tuberculosis or mild chorea, that has escaped the attention of parent, or even if the parents *do know* the child is half sick they send them to school to get them out of the way, or from fear of the "delinquent officer."

There are many sound reasons why, in this day and age, there should be "medical inspection in our schools." Some are so apparent that it is useless to mention them. The more prominent are, viz: First, that which is embodied in what, for convenience, we call "Preventative Medicine." Medical examination of the school children would bring to light many physical abnormalities which, if corrected, would control the whole child-life, and to some extent the future destiny of the community at large, for healthy children are quite apt to grow up to be strong, and useful men and women.

In a recent paper before this "academy" I made the statement (based upon statistics and experience) that at least 75 per cent. of cases of deafness are curable; or, in other words, are "preventable" if taken at the proper time. This fact is worth considering from an economic point of view. I think you medical men will subscribe to the truth of the statement, when I say that at least one in five of school children will be found to be mouth-breathers, because of nasal disorders or large tonsils or adenoid growths. As these conditions are known to be the direct contributing cause of deafness, discharging ears, and predisposed to nervous trouble, and through infection to rheumatism and even tuberculosis, it brings immense responsibilities upon the Board of Education and the medical fraternity alike.

The public have become fairly well informed as to the importance of regular examination of the eye-sight of the school children, and in our city schools an attempt has been made to bring this about. Each teacher is supposed to be supplied with a "Snellen" chart (but generally only one chart in whole school) and each pupil's visual power is supposed to be obtained and recorded, and if distinctly below the normal, the parents of the child or the sanitary officer is notified that an oculist should be consulted. Provision is made in our own city, as you all know, whereby the poor may have the services of the oculist gratuitously, a very nominal charge made for glasses furnished, which are paid for by the Board of Education.

This is very commendable, as far as it goes, but the experience of the oculists in our city shows that only a very small per cent. of these defective eye-sight children ever reach them.

It is a conservative estimate that one in three children *have trouble,* either with their eyes, nose or throat that need attention; and it is the conviction of teachers, and the medical profession, that *something* should be done to bring this about, if we hope to obtain the best possible conditions, physically and mentally, for the present rising generation. The above assertions that I have made might give you the impression that I believed that all that is required is medical supervision of the schools by a specialist—*far from it.* The best benefits can only be accomplished by union of the two forces, viz: There should be an internist and specialist for each school. Of the two I am convinced that the physician's duties are of equal if not more importance than the specialist, certainly more diversified. He should guard the school child from the evil effects of a good, *but many times,* abused compulsory educational law. In the discussion of Dr. Nobles' paper at our last meeting I was impressed with the fact that, beyond doubt, many children are kept, from fear of the authorities, in school who from physical or nervous defects have no right to be there and are receiving little but physical injury and mental discouragement by their school life. Others, classed as precocious children, are pushed ahead, making grades in an incredulous short time, simply because the teacher is over-zealous and the parents are desirous of demonstrating the extraordinary ability of their child, and from indoor confinement and over-study are driven into inevitable nervous ruin, and later mental incapacity. This cramming method, so much in vogue in our schools, at the expense of the pupils health, should be discouraged, and the state should be bound to take cognizance of the physical welfare of its school children first, last and all the time.

The second reason for medical inspection of our schools is found in the extension of our school system.

In former years the schools were widely scattered and irregularly attended, the terms were short, and there seemed no special need of hygienic attention. A half century ago we were a set of rural communities, now we are an urban nation, over 35 per cent. live in cities, and many of the residents of the country send their children to the city schools to be educated. This fact has rendered essential greater attention to water supply, problems of light and air in public school buildings, the isolation of contagious diseases, and a thousand other matters of greater or less importance, but apparently less needed in a rural community.

Incidentally I might mention it is a sad fact that the inhabitants of the country, school *authoratives* or otherwise, wilfully ignore, or are ignorant of the simplest sanitary and hygienic measures.

It is right here in these country schools that the health of the children is shamefully neglected. Fresh air is so free and plentiful that its value is not realized, and it is safe to say that the majority of all inhabitants, intelligent or otherwise, sleep in stuffy bedrooms and live in ill-ventilated rooms, thereby inviting catarrhal troubles and various other ailments. Adenoid growths and hypertrophied tonsils are alarmingly common in our rural districts. The *schools are full of them,"* is what a sanitary inspector told me the other day. She brought one of them to me for operation the other day, and said that there were three more in the same family that ought to be operated on, but the parents would not consent to bringing only the one that is getting deaf. They said the others would outgrow it. What folly, because the damage is done long before the growths atrophy. How to arouse the apathy of the public on a "good-health crusade" is one of the difficulties of the day. It never will be done until the state makes it compulsory, as vaccination, school attendance, etc. Says Dr. Wm. H. Allen, Secretary of the Bureau of Municipal Research, "The obligation between the State and the child is a reciprocal one, and when the State for its own protection compels a child to go to school, it pledges itself not to injure itself by injuring the child." Again, to quote from the British Board of Education: "Medical inspection is founded on the close connection which exists between the physical and mental conditions of the children, *and the whole of education.* It seeks to secure ultimately, *for every child, normal or defective,* conditions of life compatible with that *full* and *effective* development of its organic functions, its special senses and its mental powers, which constitute a true education."

That this medical inspection of our schools has come to stay is conclusively shown by the below statements. Twelve states have taken legislative action, either compulsory or permissive. Of these the best progress has been made in the North Atlantic and Western division of States, where 60 per cent. of the cities have taken it up. The poorest showing is made in the Southern States, where only about 20 to 30 per cent. of the cities have medical inspection of their schools. In 75 per cent. of the cities the work is prosecuted under the Board of Education.

Of 758 cities tabulated, 337 have systems of medical inspection. 301 have inspection for the detection of contagious diseases. 167 cities have physical examination of school children, most of them not only when they enter, but at stated periods. In 187 cities vision and hearing tests are conducted by the doctor. In 399 cities vision and hearing tests are conducted by teachers. There are 1194 school physicians employed as permanent members of educational forces. 371 nurses are employed in 76 cities. 48 cities have school dentists. About 25 cities are supporting open air schools, and according to Dr. Straw of New Hampshire, from whose article the above statistics are taken, no failure has been recorded. He says the children gain in weight, work less, play more, and progress faster than those in ordinary schools. He says 97 cities give special care and instruction to all school children found predisposed to or are already infected with tuberculosis and provide out-door schools for them.

New York City last year made over and equipped twenty school rooms, in regular buildings, for the better care of the sick and well school children, besides establishing a number of independent schools for out-door instruction.

That progress is being made in endeavoring to better the physical as well as the mental condition of our school children is in evidence from all sources. Only a few years ago medical inspection meant a hurried looking over of school children to discover measles, scarlet fever, diphtheria, etc. Now most of the cities look more for defective vision or anaemia, incipient tuberculosis, etc. Not long since adenoid growths were almost unheard of by school teachers. Now 171 cities make examinations for adenoids and hypertrophied or diseased tonsils, for they are known to be a more serious menace to a healthy development and school pregress than most anything else.

It is apparent from all standpoints that medical inspection of our schools is a wise and much needed condition, and is well worth our serious consideration. But how to bring it about is the perplexing problem. That the teachers should be required to make the examinations is an injustice for two main reasons; first, it adds to their already overburdensome responsibilities; and, second, since the tests, to be of any real value, require technical skill. In many of the cities a qualified school nurse is employed to supplement the work of the physician, and has been found a most excellent expedient.

To properly carry out school inspection in all of its many details requires a broad and practical knowledge of hygiene, which includes lighting, heating, ventilation, drainage, disinfection, as well as judgment of the physical endurance of the child from a medical standpoint. Specialists must examine into the visual power of the child and also determine whether deafness exists, and its cause. If the child is backward in his or her studies, it certainly should receive especial attention from both specialist and physician. Both Philadelphia and Chicago have a private pathological clinic for such children, and its duty is to inquire into nervous difficulties, hereditary troubles or other deep-seated defects. The following is a list of questions and answers indicative of the extent and purpose of the work of medical inspection of schools. These have been adopted as a standard by some cities:

1. Who and what should be physically examined? Answer. All children, normal students, teachers, janitor, buildings, grounds in all school districts, public, parochial, private, rural and urban.

2. How often? Answer. At least once a year.

3. How many children need treatment? Answer. Seven out of ten; three out of ten for eyes; two out of ten for breathing troubles; seven out of ten for bad teeth.

4. What is the penalty for physical defect? Answer. Retardation, discouragement, dropping out of school, annual waste estimated up into the millions.

5. Does examination lead to treatment? Answer. Yes, in nine cases out of ten, if the parents understand properly.

6. How should medical inspection be administered? Answer. As now only about one-quarter of the cities are under the Board of Health, and three-quarters are under the Board of Education, it would seem that the latter should control it.

7. Should the work be done by the principals and teachers, properly qualified nurses or by physician? Answer. The most excellent expedient has been to employ nurses to supplement the work of the physician. The principals of the schools with their corps of teachers are more than willing to lend their assistance and their usefulness can hardly be estimated as an aid to this work.

---

RADICAL REMOVAL OF OESOPHAGEAL CANCER. Willy Meyer, at the recent meeting of the Am. Gastro-enterologic Assn., stated that two operations, thus far successful, had been reported during the year, depending upon the use of negative pressure apparatus. Bloodgood stated that a third case had recently been reported.

---

DANGER OF REMOVAL OF PIECE OF CANCER FOR DIAGNOSIS. Bloodgood of Baltimore, at the recent meeting of the Am. Gastro-enterologic Assn., cited the well known fact that removal of a section of cancer from the tongue for examination, was almost always followed by metastasis whereas cases radically operated, even at a later stage, had a fairly good prognosis. He pointed the moral as applied to the use of forceps through the oesophagus and with regard to cancer generally.

---

RURAL ORIGIN OF TYPHOID. The *Jour. of the A. M. A.*, April 12, 1913, comments editorially on Boldman's study of conditions in New York City, and his conclusion that over half the cases are due to out-of-town infection. In 1907, we stated in a discussion at the A. M. A. meeting a similar conclusion for Buffalo, this conclusion being, apparently, unanimously disapproved. In 1908, in Paris, where a separate supply of potable water is piped from carefully guarded springs, typhoid is almost unknown except as the cases are brought in from the suburbs or as the infection is brought in from without by residents.

---

CALCULUS OF ANTRUM OF HIGHMORE, Norman B. Carson of St. Louis, *Interstate Med. Jour.*, March, 1913, reports a case with swelling of the cheek and exophthalmus in a man aged 67. A squamous epithelioma was removed and also a black, shell-like calculus, ⅛ inch in diameter, the interior of the calculus being light yellowish and coarsely granular. It consisted almost entirely of calcium phosphate.

